# Epidemiology of burns in a teaching hospital in south India

**DOI:** 10.4103/0970-0358.41108

**Published:** 2008

**Authors:** R. Raja Shanmugakrishnan, V. Narayanan, P. Thirumalaikolundusubramanian

**Affiliations:** Departments of Medicine and Plastic Surgery, Madurai Medical College, Madurai - 625 020, Tamil Nadu India

**Keywords:** Body surface area of burn, epidemiology of burns, first aid, south India

## Abstract

Despite many medical advances, burns continue to remain a challenging problem due to the lack of infrastructure and trained professionals as well as the increased cost of treatment, all of which have an impact on the outcome. There is very little information on the pattern of outcomes among burn patients in relation to clinical aspects in India. Hence, the present study was undertaken in a burns unit to determine selected epidemiological variables, assess the clinical aspects (etiology, extent and anatomical location) and first aid measures adopted and finally to analyse the outcomes in cases of burn injuries. In addition, we have sought to suggest measures to remove myths about pre-hospital burn treatment and provide recommendations to healthcare professionals.

## INTRODUCTION

Despite many medical advances, burns continue to remain a challenging problem due to the lack of infrastructure and trained professionals as well as the increased cost of treatment, all of which have an impact on the outcome. Previous epidemiological studies from different parts of India[1–6] have revealed that burn cases are prevalent all over the country. Most of these patients are poor and hence, seek healthcare from government hospitals. There is no information on the pattern of outcomes among burn patients in relation to clinical aspects in India.[7] Hence, the present study was undertaken in a burns unit to determine selected epidemiological variables, the clinical aspects (etiology, extent and anatomical location) and the first aid measures adopted and to analyze the outcomes. In addition, we sought to suggest measures to remove myths about prehospital burn treatment and provide recommendations to healthcare professionals.

## MATERIALS AND METHODS

After obtaining Institutional Ethics Committee approval, a prospective study was carried out over a 100 day period to analyse 150 consecutive burn cases admitted to a specialist 30 bedded burns unit of a large Government Teaching hospital in South India with a total of 2200 beds. The socio-demographic, clinical and in-hospital outcomes of these burn cases along with details of treatment adopted prior to hospitalization were collected by the principal author and confirmed by the second author. The patient data was recorded for age and gender, extent of burn, etiology, method of extinguishing the flame and first aid received and finally clinical outcome in hospital. The data were entered in a Microsoft® Excel spreadsheet and analyzed using Chi Square Test.

## RESULTS

**Age and gender:** A total of 150 burn patients were treated during 100 days. There were 62 males (41.3%) and 88 females (58.7%). Their ages ranged from three to 59 years in males and four to 75 years in females. The mean (±SD) was 31.58 (±11.64) for males and 30.18 (±15.60) years for females (*P* > 0.05). However, men in the age group of 25 to 34 years and women aged 15 to 24 years suffered from burns significantly more than the other age groups (*P* < 0.05). The distribution of cases in relation to the age group and gender is depicted in Table 1. The total body surface area (TBSA) in relation to the age group is provided in Table 2. Analysis revealed that individuals belonging to the 25 to 34 years' age group suffered from burns of the highest TBSA and the mean (±SD) was 67% (±34) which was statistically significant (*P* < 0.01).

**Table 1:** Distribution in relation to age and gender

*Age groups Sex Total (in years)*	*Sex*				*Total*	
		
	*Male*		*Female*		*n*	*%*
				
	*n*	*%*	*n*	*%*		
<15	5	8.06	12	13.64	17	11.33
15-24	14	22.58	36 *	40.91	50	33.33
25-34	23 *	37.10	17	19.32	40	26.67
35-44	12	19.35	8	9.09	20	13.33
45-54	7	11.29	8	9.09	15	10.00
>55	1	1.61	7	7.95	8	5.33
Total	62	100	88	100	150	100

* *P* < 0.05 {Chi square Test} significant

**Table 2:** Age group and TBSA*

*Age group (in years)*	*Mean ± SD TBSA in %*	*Median TBSA in %*
<15	51 ± 34	55
15-24	55 ± 33	50
25-34	67 ± 34	85
35-44	47 ± 38	30
45-54	49 ± 34	45
≥55	33 ± 29	20

Significant (*P* < 0.01), *TBSA - total body surface area

**Etiology:** The etiologies were determined by personal interview with 103 of the 150 patients who were *compos mentis* during their hospital stay. This could not be done for the remaining cases as they were seriously incapacitated or died soon after admission. Analysis of flame burns revealed them to be accidental in 45 [explosion of kerosene stove in 24, burns sustained while cooking food using firewood in 10, tilting of kerosene lamp in 7, and one each due to fall in the fire-walking religious ceremony, falling into an open fire under the influence of alcohol, fire accident in brick and firecracker industries], suicidal in 25 [family problems in 18, depression due to other illnesses in six and dowry-related death in one], homicidal in 8 [five acid burns and three flame burns due to personal rivalry] and incidental in 5 while trying to save other burn victims. Scalds were noticed in 15 [due to hot oil in seven, hot food in five and hot water in three] and were due to accidental reasons. Even though another 47 victims had flame burns, their attributes could not be ascertained in view of the reasons stated above. The details in relation to 103 patients and their age groups are provided in Table 3.

**Table 3:** Causes of burn

*Age group*	*Accidental*		*Suicide*		*Homicide*		*Incidental*		*Scalds*		*Electric*		*Total*
							
	*n*	*%*	*n*	*%*	*n*	*%*	*n*	*%*	*n*	*%*	*n*	*%*	
<15	8	17.8	4	16	0	0	0	0	4	26.6	0	0	16
15-24	15	33.3	11	44	3	37.5	3	60	5	33.3	0	0	37
25-34	12	26.7	4	16	0	0	2	40	0	0	4	80	22
35-44	2	4.4	3	12	3	37.5	0	0	2	13.3	1	20	11
45-54	4	8.9	3	12	2	25	0	0	1	6.6	0	0	10
>55	4	8.9	0	0	0	0	0	0	3	20	0	0	7
Total	45	100	25	100	8	100	5	100	15	100	5	100	103

**How was the fire extinguished?** The methods practised to quench the fire at home were pouring water in 55%, covering with bed sheets or gunny bag in 35%, making the victim roll on the ground in 15% and throwing sand over the victim in 4% or a combination of the above.

**First aid:** A total of 83 (55%) patients were brought directly to the hospital within three hours. They were immediately resuscitated with ringer lactate according to the Parkland formula and given intravenous antibiotics and ranitidine. The remaining consulted their local doctors and were provided with intravenous fluids in 49% (33 patients), analgesics in 40% (27 patients) and ointments in 17% (11 patients) or a combination thereof. 19% didn't receive any treatment from the local doctor when approached for professional assistance other than referral to specialist centres. Unfortunately, three doctors advised the relatives to apply liquid ink used for fountain pens or herbal extracts.

**Clinical outcome:** Of the 150 patients, 86 died with an overall mortality of 57.33%. It was found that none of the cases with <30% TBSA burns died while all those with >55% TBSA burns died; the outcome of patients with 30-55% TBSA burns was variable. The patients with TBSA of 81 to 100%, 61 to 80%, 41 to 60% and 31 to 40% died within two, four, six and eight days respectively. The details are provided in Table 4. Thus, the TBSA was directly related to the death rate whereas it was inversely related to the days of survival.

**Table 4:** Distribution of cases and death in relation to TBSA and hospital stay

*% of burns*	*Number of cases*	*Number of deaths*	*Average number of days of survival*
<21	40	0	0
21-40	26	7	8.5
41-60	22	17	6.4
61-80	14	14	3.9
81-100	48	48	2

## DISCUSSION

Understanding the epidemiological aspects and clinical details is helpful to find out the lacunae in burns' treatment and the need to improve the same. In the present study, 150 cases of burns were hospitalized over a period of 100 consecutive days in a 30 bedded burns unit of a large multispecialty teaching hospital with 2200 beds in Madurai.

Eighty six out of 150 patients died and the in-hospital mortality was 57.33% which is consistent with the series of Subrahmanyam[1] (56.5%) in Solapur, Maharashthra, Bilwani *et al*.[3] (58.26%) in Ahmedabad, Gujarat and Jayaraman *et al*.[8] (52.33%) in Chennai, Tamil Nadu. It was lower than the observations of Puri[9] (90.2%) in Pune, Maharashtra but higher than that of Gupta *et al*.[10] (48.33%) in Jaipur and Sarma *et al*.[11] (18.3%) in Digboi, Assam. The low mortality rate in Sarma's series could be attributed to a higher proportion of industrial accidents and lesser homicidal and suicidal patients. The higher mortality rate in the present series could be attributed clinically to burns involving more than 55% TBSA and to nosocomial infections among others.

Among women, 40.9% of the victims belonged to the age group of 15 to 24 years[2] and the triggering factor for burns were young age at the time of marriage combined with inability to cope with the physical and psychological stress of marriage,[2,6,12,13] harassment from parents-in-law, inadequate precautions during cooking and wearing of the loose Indian sari.[3] In contrast, 37% of men belonged to the age group of 25 to 34 years and the factors attributed to burns were unemployment, depression and stressful situations. TBSA observed among the age group 25 to 34 years was the highest and was due to flame burns. Hence, burns among them were likely to be intentional. Analysis of death in relation to TBSA and the number of days survived among those who died, revealed that those with TBSA of 81 to 100%, 61 to 80%, 41 to 60% and 31 to 40% expired within two, four, six and eight days respectively. As all those with burns of TBSA < 30% survived and those > 55% died, it is considered that the vulnerable group were those with burns of TBSA between 30 to 55% who need more care and support to overcome multiple internal and external factors contributing to the mortality. Hence, in the event of mass casualty due to burns or in areas/hospitals with suboptimal facilities, we suggest that priority be given to those patients with burns of TBSA of 30 to 55%.[14]

Flame burns accounted for 86.6% of all cases. Although accidental flame burns was the most common cause (57%), it was far less when compared to the series of Subrahmanyam (80%) and Bilwani *et al.* (77%). This variation may be attributable to the socio-environmental factors discussed earlier.

Kerosene stoves contributed to 48% of accidental burns when compared to 2% due to gas stoves. As most of the kerosene stoves are of inferior quality,[5] the occurrence could be greatly reduced by increasing consumer awareness, enforcing quality standards for stoves or replacing kerosene stoves by gas stoves. Burns due to industrial accidents were far less encountered during the study and observed in only two cases - one from the brick industry and the other from the fire cracker industry. The reason for the low prevalence of industrial accidents was due to the predominantly agrarian rather than industrial population around the study area. Scalds were observed in the “extremes of age” group because of the carelessness and restlessness associated with children and decreased mobility and slow reflexes in the geriatric population. The critical area of involvement of burns observed was face (58%), neck (68.7%), hands (66%) and multiple areas in many cases, which enhances the work load on the unit caring for those who survived. Although pouring water is the best way to quench the fire, it was known and practiced in only 55% of the cases. In this study, the practice of other conventional methods had resulted in deep burns and contributed to high morbidity and mortality. Many patients and their relatives in this group actually believed that pouring water was harmful to the patient.

As burns are very frightening and catastrophic, patients go to the local practitioner for first aid. When prehospital treatment was analyzed, 83 patients were referred immediately due to the severity of burns and the rest were given substandard care. It was disappointing to note that doctors too practiced unconventional techniques (fountain pen ink and herbal preparations) as first-aid measures to treat burn victims. As their treatment modalities reflected their suboptimal knowledge of treatment of burns, these doctors require continuing medical education.[15] It is also recommended that educational authorities should post interns during their undergraduate education to burns wards for at least seven days to learn the basics of burn injuries and management of the same. The study revealed that burn surgeons have to initiate efforts to educate the public and health professionals regarding first aid for burns and to remove the myths about burn treatment. The importance of pouring water immediately to quench the fire on the victim should be communicated to all through mass media.

The strengths of this study were the prospective nature of the study, evaluation of individual cases by a single person (first author), confirmation by the second author and follow-up of the patients.
